# “Sounding Black”: Speech Stereotypicality Activates Racial Stereotypes and Expectations About Appearance

**DOI:** 10.3389/fpsyg.2021.785283

**Published:** 2021-12-24

**Authors:** Courtney A. Kurinec, Charles A. Weaver

**Affiliations:** Department of Psychology and Neuroscience, Baylor University, Waco, TX, United States

**Keywords:** stereotypes, social categorization, race, Black Americans, phenotype, dialect, speech perception

## Abstract

Black Americans who are perceived as more racially phenotypical—that is, who possess more physical traits that are closely associated with their race—are more often associated with racial stereotypes. These stereotypes, including assumptions about criminality, can influence how Black Americans are treated by the legal system. However, it is unclear whether other forms of racial stereotypicality, such as a person’s way of speaking, also activate stereotypes about Black Americans. We investigated the links between speech stereotypicality and racial stereotypes (Experiment 1) and racial phenotype bias (Experiment 2). In Experiment 1, participants listened to audio recordings of Black speakers and rated how stereotypical they found the speaker, the likely race and nationality of the speaker, and indicated which adjectives the average person would likely associate with this speaker. In Experiment 2, participants listened to recordings of weakly or strongly stereotypical Black American speakers and indicated which of two faces (either weakly or strongly phenotypical) was more likely to be the speaker’s. We found that speakers whose voices were rated as more highly stereotypical for Black Americans were more likely to be associated with stereotypes about Black Americans (Experiment 1) and with more stereotypically Black faces (Experiment 2). These findings indicate that speech stereotypicality activates racial stereotypes as well as expectations about the stereotypicality of an individual’s appearance. As a result, the activation of stereotypes based on speech may lead to bias in suspect descriptions or eyewitness identifications.

## Introduction

Every day, we interact with those we do not know in order to perform our jobs, run our errands, or engage in other, more leisurely activities. Making use of the available social information, we quickly form impressions about these unfamiliar people and use those impressions to guide our interactions. For instance, we may use the available cues to make assumptions about another person’s membership in certain social categories, such as their likely gender, ethnic group, occupation, or social status, and the stereotypes or beliefs we associate with those social categories influence the traits we expect this person to possess.

How strongly we link stereotypes for a social category to a specific individual often depends on the extent to which that individual is seen as a typical member or exemplar of that group. Individuals who possess more of the features related to their social group are often more closely associated with stereotypes about that group (Blair et al., 2004b; Walker and Wänke, 2017). Unfortunately for Black Americans, these stereotypes include expectations about criminality (Eberhardt et al., 2004) and may influence how more stereotypical Black Americans are perceived and treated by the legal system. More stereotypically Black individuals are more likely to be associated with crime or a criminal label both by members of the general public and police officers (Eberhardt et al., 2004; Kleider et al., 2012) and are more likely to be misidentified as a suspect by eyewitnesses (Knuycky et al., 2014; Kleider-Offutt et al., 2017). The influence of stereotypicality extends to sentencing, such that when the victim is White, Black defendants with more stereotypical features are more likely to be given the death penalty than less stereotypical looking Black defendants (Eberhardt et al., 2006).

Studies investigating stereotypicality often focus on physical characteristics; for example, those with fuller lips or a wider nose are more likely to be viewed as phenotypically Black (Blair et al., 2002, 2004b; Hagiwara et al., 2012). These Afrocentric facial features can be used separately or in combination with skin tone to influence judgments about race typicality (Stepanova and Strube, 2009, 2012b; Dunham et al., 2015). However, judgments about individuals are influenced by more than their static visual appearance; indeed, some of our interactions do not include visual information, e.g., telephone calls or online voice chat. It is unclear how other aspects of an individual—specifically how they speak—contribute to judgments of whether an individual is “stereotypically Black.”

### Language as a Marker of Social Category

Language is an important tool in social categorization (Rakiæ et al., 2011b; Dragojevic et al., 2018). How a person speaks can act as an index or sign of one’s background (Bucholtz and Hall, 2005), and this indexical information about a person’s social category information is quickly and automatically accessed. For instance, information about a person’s likely gender category can be accessed around 150 ms after voice presentation (Munson and Solum, 2010). In some cases, the way a person talks can be an even more important indicator of the social category to which an individual belongs than facial features (Rakiæ et al., 2011a).

Social category information obtained from a person’s speech can activate stereotypes or other assumptions not only about the speaker’s linguistic background, but also the social groups to which they likely belong (Giles and Rakiæ, 2014), providing a gateway for individuals to make judgments about the speaker (Giles, 1970; Mulac and Rudd, 1977). Importantly, the stereotypes and attitudes activated in relation to a given speaker depend heavily on the listener. The stereotypes a listener associates with a specific social group are dictated by that listener’s social environment and cultural context, to include both implicitly and explicitly held beliefs, as well as the listener’s ability to notice and classify certain linguistic features, the listener’s expectations about the conversation, and their own communication goals (Cargile and Bradac, 2001; Preston, 2018).

One cultural factor that shapes how speakers are perceived is the level of standardization of the dialect they employ. Dialects, or the way of speaking associated with a certain regional, cultural, or ethnic group, are often described as either “standard” or “non-standard,” with standard variants of a language being ones that are supported by the state and/or other influential institutions (Milroy, 2001; Milroy and Milroy, 2012). Accordingly, the identities and dialects of those in power influence which dialects are considered standard (Lippi-Green, 1997). Standard dialects, such as General American English in the United States (U.S.) or Received Pronunciation in the United Kingdom (UK), are often viewed more favorably and seen as more prestigious than non-standard dialects (Dent, 2004; Morales et al., 2012), even by speakers of non-standard dialects (Anisfeld et al., 1962; Carter and Callesano, 2018). How those who use non-standard dialects are viewed often depends on how the people most closely associated with that dialect are perceived. For instance, people from the Southern U.S. are stereotyped as being uneducated, poor, and lazy (Slade and Narro, 2012). Unsurprisingly, speakers using a Southern U.S. dialect, a non-standard dialect of American English associated with this region, are seen as less wealthy, less intelligent, less healthy, and less attractive than speakers using a more standard American dialect (Dent, 2004; Phillips, 2010; Shamina, 2016). Further, linguistic features associated with Southern U.S. English are implicitly associated with blue collar jobs and lower intelligence (Campbell-Kibler, 2012; Loudermilk, 2015). In this way, judgments about speakers of a given dialect can reflect the stereotypes about members of that social group.

### Sounding Black

Given the interconnectivity between language, social categorization, and stereotypes, it is likely that individuals who “sound Black” are more likely to be identified as Black Americans and therefore more likely to be associated with stereotypes about the group. One way individuals may be thought to “sound Black” is through their use of African American Vernacular English (AAVE). AAVE is a non-standard dialect of American English closely associated with and spoken predominantly (but not only) by Black Americans (Cutler, 2003; Rickford, 1999). Often denigrated as slang or improper English, AAVE is in fact a valid language system, with regular phonological and grammatical features such as -ing dropping (e.g., “goin”’ vs. “going”), *r*-lessness (e.g., “fo”’ vs. “four”), negative concord (e.g., “He ain’t seen nothin”’), and the use of habitual be (e.g., “She be workin”’ indicates “She’s often working”) (Pullum, 1999; Thomas, 2007; see Jones, 2015 for more on regional variations in AAVE). Like speakers of other non-standard dialects, speakers of AAVE are seen less favorably than speakers of the more standard General American English in most contexts (Payne et al., 2000; Koch et al., 2001; Dent, 2004; Rodriguez et al., 2004; Billings, 2005). Speakers of AAVE are seen as less competent, less sociable, less professional, less educated, and of poorer character than speakers of more standard American English (Payne et al., 2000; Koch et al., 2001; Dent, 2004; Billings, 2005). As with the Southern U.S. dialect, many of the traits associated with AAVE are also associated with its dominant speakers: Black Americans (Devine and Elliot, 1995; Maddox and Gray, 2002). For instance, individuals show a greater implicit association between weapons and AAVE speakers than more standard speakers (Rosen, 2017), suggesting that stereotypes about criminality and violence, often associated with Black Americans, are also linked to AAVE speakers. Further, AAVE’s close association with Black Americans has also led to linguistic profiling, or discrimination against those who speak a certain way due to their assumed membership in a social group. Discriminating against someone for their way of speaking can allow for anti-Black bias to circumvent legal protections, leading to worse outcomes and fewer opportunities in areas such as housing for those who use AAVE and are assumed to be Black (Purnell et al., 1999; Massey and Lundy, 2001).

However, not all Black Americans speak AAVE, and those who do speak AAVE do not use it all the time or in all contexts (Rickford, 1999; McCluney et al., 2021). Yet listeners can reliably identify the race of Black speakers regardless of dialect with over 85% accuracy in longer (10 second) clips (Kushins, 2014) and approximately 60–70% accuracy after listening to a one-second clip or a single word (Walton and Orlikoff, 1994; Purnell et al., 1999). This ability to quickly identify the racial identity of a speaker is likely due to the presence of certain phonological features, e.g., final consonant dropping or vowel quality (Walton and Orlikoff, 1994; Thomas and Reaser, 2004; Perrachione et al., 2010), but there is no consensus on which specific linguistic cues trigger the perception of a speaker as Black. Despite this lack of scientific consensus, it is likely that listeners have learned, through their social or cultural environment, to associate certain linguistic features with Black Americans and use those cues to identify speaker race (Perrachione et al., 2010). As a result, Black speakers who do not employ the expected linguistic features can be miscategorized as members of other races or ethnic groups (Thomas and Reaser, 2004; Perrachione et al., 2010).

Regardless of whether listeners are picking up on AAVE or other linguistic features associated with Black Americans, the strength with which speakers employ these features likely predicts whether listeners will categorize speakers as Black and the stereotypes assigned to them. Rodriguez et al. (2004) found that speakers who had a stronger AAVE dialect (i.e., used more AAVE features) were rated less favorably than those with a more moderate AAVE dialect. Thus, one would expect that speakers who have stronger dialects and sound “more Black” to listeners will be not only more likely to be identified as Black, but also will be more associated with stereotypes about Black Americans—including expectations about criminality and violence. However, this has yet to be directly investigated.

If sounding “more Black” does lead to an increase in Black stereotypes, it could also lead to the assumption that the speaker has a more stereotypically Black appearance as well. Previous work has found that stereotypes can influence expectations about appearance. Hughes and Miller (2016) found that, in line with the “what sounds beautiful is good” stereotype (Zuckerman and Driver, 1989), individuals with more attractive voices are expected to have more attractive faces. Separately, Osborne and Davies (2013) found that participants who watched a video of a crime stereotypically associated with Black people remembered the perpetrator as appearing more phenotypically Black than those who watched the perpetrator commit a crime stereotypically associated with White people, even when the crimes were matched on severity and violence. Being perceived as sounding more stereotypically Black could similarly activate listeners’ stereotypes about Blackness and influence the expectations a listener has for their appearance—a supposition with critical implications for the legal system, e.g., in ensuring reliable suspect identifications.

Given that linguistic profiling and discrimination based on how a person speaks are not explicitly prohibited under U.S. law (Wiehl, 2002; MacNeal et al., 2019), understanding how speech stereotypicality influences assumptions about a speaker is needed before any countermeasures to minimize linguistic bias can be developed. To address this issue, we conducted two experiments to investigate the relationship between sounding more stereotypically Black and the assignment of stereotypical traits associated with Black Americans (Experiment 1) and decisions about likely appearance (Experiment 2). In both experiments, participants listened to audio recordings from Black speakers before making their judgments. We expected that speakers whose speech is perceived as more stereotypically Black would be associated with more stereotypical traits about Black Americans and with a more phenotypical Black appearance.

## Experiment 1

Those who are perceived as looking more phenotypically Black are also more likely to be associated with stereotypes about Black Americans (Blair et al., 2004b). In Experiment 1 we explored whether this pattern extended to those who are perceived as *sounding* more Black. Participants listened to and evaluated audio recordings taken from the internet of American and British Black male speakers. We had two hypotheses for this experiment. First, we expected that participants would assign more stereotypical traits to speakers they rated as sounding more stereotypically Black. Second, since listeners rely on learned linguistic cues to identify Black speakers, we anticipated that our U.S.-based listeners’ concept of “stereotypically Black” would be informed by their cultural context. Thus, they would assign more stereotypes to more stereotypical-sounding Black speakers who were also perceived as American.

### Materials and Methods

#### Participants

We recruited 75 participants from Amazon’s Mechanical Turk (MTurk) using the TurkPrime interface (Litman et al., 2017). Only U.S.-based workers who had completed at least 100 Human Intelligence Tasks (HIT) and who had HIT approval rates of 98% or greater were allowed to participate in this study. Workers were paid $0.50 for approximately 10 min of work. The sample was predominantly female (62.7% women; 34.7% men; 1.3% gender neutral; 1.3% prefer not to answer) and White (69.3% White; 5.3% Black; 8.0% Asian; 5.3% Hispanic or Latino/a; and 12.0% multiracial), and the mean age of the sample was 39.77 years (*SD* = 12.72; Range = 20–71). Participants were overall well-educated; 45.3% of the sample reported they had a bachelor’s degree, and 26.7% reported they had at least some college credit. Only 2.7% reported they did not possess at least a high school diploma or its equivalent.

Participants used a variety of terms when freely describing their own dialects, with the most common labels being some derivative of “American English” (13.3%), “Midwestern” (13.3%), or “Standard American” (10.7%). Other expected regional (e.g., “American East Coast,” “Bostonian,” “Texan,” or “Southern English”) and racial/ethnic terms (e.g., “African American,” “Chinese English,” or “Italian American”) also appeared. Interestingly, some participants labeled their dialects as “White” or “Caucasian” (6.7%).

In order to minimize low-effort or bot responses, we removed four participants’ data for providing nonsensical or off-topic responses (e.g., “NICE”) to our free response dialect question. Other responses that were related to a way of speaking but did not describe a dialect *per se* (e.g., “slang” or “soft spoken”) were retained in analyses, leaving us with data from 71 participants (675 observations). According to G*Power (Faul et al., 2009), *a priori* power analysis for a two-tailed multiple regression with our seven predictors would require a sample size of at least 55 to detect an interaction of medium effect size (*f*^2^ = 0.15), with α = 0.05 and 1-β = 0.80. Using these same parameters for α and 1-β, our sample of 71 would allow us to detect at least an effect size of *f*^2^ = 0.11.

#### Materials

##### Audio Recordings

We created 20 audio recordings featuring 14 Black North American male and 6 Black British male speakers. To have more ecologically valid recordings, the audio was taken from YouTube videos by searching terms such as ‘‘Black British,’’ ‘‘Black English,’’ and ‘‘Black American.’’ The audio was then shortened to a sample of the speaker’s speech. These clips ranged between 18 and 35 s due to variations in the length of speakers’ utterances. In the clips, speakers discussed a variety of topics, i.e., travel experiences (*n* = 3), restaurants/food/diet (*n* = 7), domestic/foreign culture (*n* = 5), working abroad (*n* = 2), creating a better life for one’s family (*n* = 1), comedians (*n* = 1), and sport (*n* = 1)^1^. The audio clips were screened for any explicit mentions of the speaker’s race or national origin. After data collection, one of the British speakers (who discussed sports) was revealed to be an American actor; as a result, this speaker was removed from analyses, leaving us with data on 19 speakers. Although we could reasonably assume the rest of our British speakers were from the UK due to the content of either the full video or their profiles, we could not find information about the nationality of two of the American speakers. Due to their use of American English dialects, we assume these speakers are from the U.S.; however, we recognize that some of these speakers could be Canadian given the overlap between Canadian and American English (see Labov et al., 2008).

##### Stereotypical Traits

To rate the speakers from the voice clips, participants were shown a list of 30 adjectives taken from Devine and Elliot (1995) and Maddox and Gray (2002). The adjectives included those associated with Black American stereotypes (athletic, criminal, lazy, poor, rhythmic, uneducated, unintelligent, hostile, loud, dirty, inferior, ostentatious, sexually aggressive, and aggressive), as well as counter-stereotypic (intelligent, kind, educated, motivated, and wealthy), and neutral adjectives (attractive, bad attitude, self-assured, unattractive, superstitious, naïve, unreliable, talkative, materialistic, arrogant, and ambitious). To create our dependent variable, we calculated the proportion of stereotypical adjectives out of all the adjectives a participant assigned to a given voice.

##### Speaker Perceived Demographics

Participants rated the speakers’ perceived race, nationality, age, voice attractiveness, speech stereotypicality, and dialect. Participants indicated the speaker’s perceived race from a list of five races/ethnicities: White/Caucasian, Black/African American, Hispanic or Latino/a, American Indian/Alaska Native, or Native Hawaiian/Other Pacific Islander; participants could also indicate “Other” and enter their own label. For perceived nationality, participants were shown five major English-speaking countries: the United Kingdom, the United States, Canada, Australia, or New Zealand. Once again, participants could also write-in an alternate answer. The speaker’s perceived age (in years) was indicated by entering a number. Perceived voice attractiveness and stereotypicality were both rated on 7-point Likert-type scales (1 = Not at all attractive/stereotypical; 4 = Neutral; 7 = Extremely attractive/stereotypical). Speech stereotypicality was rated in terms of the perceived race of the speaker; for example, if the participant believed the speaker’s race was White/Caucasian, the speech stereotypicality question asked them how stereotypically White/Caucasian the speaker’s voice was. Finally, participants were shown four options for perceived dialect, as well as the option to provide another response: African American English (“Ebonics”), Standard American English (“Midwestern”), Black British English, or Standard British English (“Received Pronunciation” or “Queen’s English”). The labels of African American English, Standard American English, Black British English, and Standard British English are equivalent to AAVE, General American English, Multicultural London English, and Received Pronunciation, respectively. These labels were used in lieu of the more appropriate naming conventions in order to make the dialects more easily understood by participants. Further, our use of the Black British English label was used so our U.S.-based participants, who are likely unfamiliar with Multicultural London English, would have an equivalent British racial dialect to AAVE. Participants were also asked to indicate how familiar they were with the speaker’s dialect on a 5-point Likert-type scale (1 = Not at all; 3 = A moderate amount; 5 = A great deal).

#### Procedure

After indicating their informed consent to the study procedures, all participants were assigned a subject number to safeguard their identity. At the beginning of the study, participants supplied demographic information and completed a short audio test to ensure that they were able to hear the recordings. Participants then listened to 10 of the 20 audio clips; the selection of audio clips and order of presentation was randomly determined. After listening to a clip, participants selected the adjectives that they believed the average person would use to describe the speaker. The instructions emphasized that the participant did not have to personally agree with the description. Next, participants chose the speaker’s likely race, nationality, and age and rated how attractive the speaker’s voice was. Finally, participants indicated how stereotypical the speaker’s voice was for their perceived race (as chosen by the participant) before choosing a likely dialect for the speaker and rating how familiar they were with that dialect. The audio clip remained on the screen while participants provided their ratings so participants could refer back to it. After rating all of their assigned voice clips, participants were asked to provide a term or label to describe their own dialect or way of speaking. Upon completing all study procedures, participants were thanked for their work and debriefed on the purpose of the study.

### Results

#### Data Analysis

To explore how perceived speech stereotypicality influences the traits people assign to a speaker, we ran a mixed effects model predicting the proportion of stereotypical traits assigned. Due to the aforementioned overlap between U.S. and Canadian speakers, we coded responses of the U.S. and Canada to the speaker’s perceived country question under the umbrella of North American (N. Am.). Therefore, the model included fixed effects for perceived race (Non-Black, Black), perceived country of origin (Non-N. Am., N. Am.), speech stereotypicality (mean-centered by participant), and their interactions, and random intercepts for participants and the individual speakers.

We also ran an additional model with perceived speaker age and voice attractiveness (mean-centered by participant) included as covariates. Speaker age was controlled for as age may moderate how stereotype content may vary not only by race, but also by the age of the individual (e.g., Andreoletti et al., 2015), which may affect both how stereotypical listeners rated speakers’ voices as well as what traits they associated with the speaker. Stereotypes about Blackness in particular may be more salient for younger rather than older Black men, as an analysis of Pennsylvania sentencing data from the late 1980s to early 1990s revealed that, controlling for crime severity and other court-related factors, young Black men received harsher sentences than older Black men and White men and White and Black women of any age (Steffensmeier et al., 1998). Voice attractiveness was included as a covariate as more attractive voices are often associated with more positive traits (Zuckerman and Driver, 1989), which may influence the adjectives listeners associate with speakers. How stereotypical a speaker sounds as a member of their perceived race and how attractive their voice is rated are likely related, given that conceptions of attractiveness broadly favor Eurocentric traits (Maddox, 2004). However, previous work investigating facial features and attractiveness found racial typicality and attractiveness had small to moderate correlations (Stepanova and Strube, 2018).

In both models, predictors were sum coded, with Non-Black and Non-N. Am. serving as the reference groups. Significance tests were run by conducting likelihood ratio tests comparing the full model to the model without the predictor of interest for all predictors. Follow-up pairwise comparisons were Bonferroni-adjusted for four tests. The data were imported into R Studio (RStudio Team, 2019) using the haven package (Wickham and Miller, 2019). Data were analyzed using the psych (Revelle, 2017), gmodels (Warnes et al., 2018), afex (Singmann et al., 2019), lme4 (Bates et al., 2015), and emmeans (Lenth, 2019) packages.

#### Speaker Perceived Demographics

##### Perceived Country

We compared speakers’ actual country of origin (N. Am. or UK) to participants’ perceived country choices (N. Am. or UK). Participants were generally able to correctly identify the country each speaker originated from, with greater accuracy for N. Am speakers than UK speakers (UK speakers 89.4% correct; N. Am. speakers 94.4% correct).

##### Speaker Perceived Race

Although all speakers were Black, participants identified speakers as Black only around two-thirds of the time (61.3%). Out of all possible racial options, participants identified our UK speakers nearly equally often as White (49.2%) or Black (44.7%), whereas our N. Am. speakers were identified primarily as Black (67.3%), with White as the second most frequent option (26.4%). A logistic mixed effects model on perceived race (Non-Black, Black) with fixed effects for speaker’s actual country (UK, N. Am.) and their perceived country (Non-N. Am., N. Am.) and random effects for participants and speakers found that neither actual nor perceived country of origin predicted perceptions of the speaker’s race (*p*s ≥ 0.240).

##### Speaker Perceived Dialect

Although our speakers used different dialects at different strengths, participants categorized UK and N. Am. speakers by region-appropriate labels. UK speakers were mostly labeled as using Standard British English (63.1%) or Black British English (29.6%), and N. Am. speakers were more likely labeled as using Standard American English (56.9%) or AAVE (35.7%).

##### Speaker Dialect Familiarity

Using the 5-point scale, participants indicated they were fairly familiar with General American English (*M* = 3.78, *SD* = 1.04, *n* = 289) and AAVE (*M* = 3.61, *SD* = 0.91, *n* = 179). As expected, participants were less familiar with Standard British English (*M* = 2.74, *SD* = 0.98, *n* = 125) and Black British English (*M* = 2.36, *SD* = 1.06, *n* = 66).

#### Proportion of Stereotypical Traits

On average, participants assigned each speaker 4.21 traits (*SEM* = 0.09). All participants assigned speakers at least one trait (Range = 1–17). Around a third of all assigned adjectives were stereotypical (*M* = 0.31, *SEM* = 0.01). The percent of each stereotypical trait assigned to speakers based on perceived country and perceived race are presented in Table 1.

**TABLE 1:** Percent of stereotypic adjectives assigned to speakers in Experiment 1, by perceived country and perceived race.

Adjective	Non-North American		North American	
	Black	Non-Black	Black	Non-Black
Athletic	**16.5**	3.9	10.6	**11.4**
Criminal	1.2	0.0	5.8	0.0
Dirty	1.2	1.0	5.2	1.9
Inferior	1.2	4.9	6.4	5.1
Lazy	3.5	3.9	9.4	6.3
Ostentatious	7.1	**12.6**	4.0	2.5
Poor	2.4	4.9	**19.5**	2.5
Rhythmic	**15.3**	9.7	15.5	6.3
Sexually aggressive	2.4	4.9	3.3	3.2
Aggressive	10.6	**12.6**	12.2	**11.4**
Uneducated	9.4	11.7	**24.9**	10.1
Unintelligent	5.9	10.7	**20.1**	**14.6**
Hostile	7.1	6.8	4.9	5.1
Loud	**22.4**	**25.2**	15.8	**20.3**

*Percentages are out of all ratings for each category. Bold indicates the top three adjectives assigned to speakers by category; four values are in bold for North American Non-Black speakers due to a tie.*

We observed a significant two-way interaction of perceived race and speech stereotypicality on proportion of stereotypical traits assigned, *B* = -0.03, *SE* = 0.01, *p* = 0.005. A test of simple slopes indicated that the slope of stereotypicality on assigned stereotypical traits was greater for perceived Black speakers than non-Black speakers, *t* = 2.83, *p* = 0.005. For perceived Black speakers, those rated 1 SD above the mean on speech stereotypicality were assigned more traits than those rated 1 SD below the mean, *t* = 2.94, *p* = 0.014. The proportion of assigned traits did not differ by rating for non-Black speakers, *t* = -1.14, *p* > 0.999, nor did it differ by race for those rated 1 SD above or below the mean (*p*s > 0.185).

The two-way interaction was qualified by a significant three-way interaction of perceived race, perceived country, and speech stereotypicality, *B* = 0.02, *SE* = 0.01, *p* = 0.027. To follow-up the three-way interaction, we conducted tests of simple slopes for the perceived race by speech stereotypicality interaction separately for perceived N. Am. and Non-N. Am. speakers. For perceived N. Am. speakers, the slope of stereotypicality on assigned traits was greater for perceived Black speakers than perceived non-Black speakers, *t* = 4.84, *p* < 0.001. Pairwise comparisons indicated that among perceived Black speakers, those rated 1 SD above the mean on speech stereotypicality were assigned more stereotypical traits than those rated 1 SD below the mean, *t* = 4.65, *p* < 0.001. For perceived non-Black speakers, this pattern was reversed; speakers rated 1 SD above the mean on speech stereotypicality were assigned fewer stereotypical traits than those rated 1 SD below the mean, *t* = –2.61, *p* = 0.037. Additionally, at 1 SD above the mean on stereotypicality ratings, perceived Black speakers were assigned more stereotypical traits than their non-Black counterparts, *t* = 3.53, *p* = 0.002, but at 1 SD below the mean perceived Black speakers were assigned fewer traits than their counterparts, *t* = –2.88, *p* = 0.017. In other words, as perceived Black American speakers were rated as more stereotypical-sounding, they were also assigned more stereotypical traits, but as perceived non-Black American speakers were rated more stereotypical-sounding, they were assigned fewer stereotypical traits. There was no such difference in slopes of stereotypicality for those perceived as Non-N. Am. speakers, *t* = –0.38, *p* = 0.704. The mean proportion of stereotypical traits assigned to speakers by perceived country, race, and stereotypicality are presented in Table 2.

**TABLE 2:** Mean proportion of stereotypical traits and standard errors for speakers in Experiment 1, by perceived country, perceived race, and stereotypicality rating.

Perceived country	Stereotypicality rating	Black	Non-Black
Non-North American	–1 SD	0.26 (0.05)	0.26 (0.05)
	+1 SD	0.33 (0.08)	0.29 (0.06)
North American	–1 SD	0.22 (0.04)	0.35 (0.04)
	+1 SD	0.39 (0.04)	0.23 (0.04)

*Standard errors of the mean (SEM) are in parentheses. Mean proportion of stereotypes is calculated for stereotypicality ratings ±1 standard deviation (SD) from the mean. Higher stereotypicality ratings indicate that speakers are more stereotypical sounding as a member of their perceived race.*

There were no significant main effects of perceived race, *B* = –0.009, *SE* = 0.02, *p* = 0.547, perceived country, *B* = –0.08, *SE* = 0.02, *p* = 0.663, nor speech stereotypicality, *B* = 0.01, *SE* = 0.01, *p* = 0.202. No other interactions were significant (*p*s > 0.691).

Perceived age was significantly correlated to sterotypicality (uncentered), although the relationship was small, *r* = 0.13, *p* = 0.001. Voice attractiveness (uncentered), on the other hand, had a small negative correlation with streotypicality, *r* = –0.15, *p* < 0.001. When adding perceived age and voice attractiveness as covariates to our model, the pattern of results remained the same. The two- and three-way interactions remained significant, *B* = –0.02, *SE* = 0.01, *p* = 0.045; and *B* = 0.02, *SE* = 0.01, *p* = 0.007, respectively. Only voice attractiveness ratings predicted proportion of stereotypical traits, *B* = –0.07, *SE* = 0.01, *p* < 0.001. As perceptions of voice attractiveness increased, the proportion of stereotypical traits decreased. Age did not have a significant effect, *B* = 0.0005, *SE* = 0.002, *p* = 0.793.

### Discussion

The findings from Experiment 1 provide support to our two hypotheses. First, speakers who were perceived as sounding more stereotypically Black were assigned more stereotypes associated with Black Americans compared to those who were perceived as less stereotypically Black sounding. Second, the relationship between sounding stereotypically Black and the assignment of Black stereotypes was true only for those seen as Black Americans. For those perceived as North Americans, more stereotypically sounding Black speakers were assigned a larger proportion of stereotypical traits than less stereotypical-sounding Black speakers. Interestingly, this pattern was the opposite for those perceived as non-Black speakers: more stereotypically sounding non-Black speakers were assigned fewer traits than less stereotypical-sounding non-Black speakers. We did not observe any differences in how speech stereotypicality affected the assignment of traits for those not perceived as North Americans. Notably, the observed pattern results remained when perceived age and voice attractiveness were added to the model. Although voice attractiveness did significantly predict the proportion of stereotypic traits assigned, it was not strongly correlated with perceived stereotypicality, echoing previous findings investigating race-related facial features (Stepanova and Strube, 2018).

It is unclear from this study why non-Black American speakers would be assigned more Black stereotypes as they sounded less stereotypically non-Black. This may represent a sort of black sheep effect (Marques et al., 1988), where our mostly White listeners are more biased against perceived non-Black (predominantly identified as White) speakers who do not conform to their expected speech. However, future research is needed to determine whether this pattern replicates when potential confounds, such as the option to identify speakers as belonging to races other than White or Black, are minimized. Regardless, our findings regarding Black American speakers are consistent with previous work on racial phenotypes. Like those seen as phenotypically Black, those who are rated as more stereotypically Black (American) sounding are associated with more stereotypes about Black Americans. Additionally, as these stereotypical traits are specific to Black Americans, listeners use vocal cues about nationality to distinguish between those from the U.S. and Canada and those from other nations.

## Experiment 2

Because we found that speech stereotypicality elicited stereotypes in a similar manner as racial phenotypes, we next investigated in Experiment 2 whether speakers who sounded more stereotypical were also expected to appear more phenotypical. After piloting audio from male Black American speakers and images of Black male faces to find high and low stereotypicality exemplars, we had participants listen to a speaker and choose which of two faces was most likely to be the speaker. We expected that participants would be more likely to choose a face that matched the stereotypicality level of the speaker’s voice; in other words, that speakers who sounded less stereotypically Black would be associated with less phenotypically Black faces and those who sounded more stereotypically Black would be associated with more phenotypically Black faces. Further, we expected that participants’ social attitudes, such as their feelings about Black Americans or their willingness to respond in a desirable manner, would be significant covariates for participants’ choice of face.

### Materials and Methods

#### Participants

Participants (*N* = 155) were recruited from Amazon’s MTurk and paid $1.00 for successful completion of the 15-min study. Only U.S.-based workers who had completed at least 100 HIT and who had HIT approval rates of 90% or greater were allowed to participate in this study, and those who participated in the pilot were not allowed to participate in this study. Five participants were removed from analyses due to failure to follow instructions, resulting in a final sample size of 150 (*M*_Age_ = 34.40, *SD* = 11.17; Range = 20–70). Participants were relatively equally divided by gender (56.7% men; 43.3% women). Participants were predominantly White (64.0%), with Black (16.7%), Hispanic and Latino/a (8.0%), Native American (5.3%), and Asian (4.7%) individuals also represented. Most participants reported having a Bachelor’s degree (46.0%), followed by those with some college (19.3%), a high school degree or equivalent (14.0%), and Master’s degree (12.7%). No participants reported having an education level lower than that of a high school degree.

To calculate an *a priori* power analysis for a two-tailed logistic regression, we used the accuracy index of 72% found by Purnell et al. (1999) as the probability that participants would choose a face that matched the stereotypicality level of the speaker’s voice. Thus, the probability of choosing a face that did not match the stereotypicality level of the speaker’s voice (*p*_1_) was 0.28. According to G*Power (Faul et al., 2009), a sample size of at least 40 would be needed to detect an odds ratio of 6.61, with α = 0.05 and 1-β = 0.80. Using these same parameters for α, 1-β, and *p*_1_, a sample size of 150 would allow us to detect an odds ratio of at least 2.59.

#### Materials

##### Stimuli

Twenty-four Black male faces and 12 Black male voices were selected based on data from a pilot study rating Black male faces and voices (see Supplementary Material). The faces were from the Chicago Face Database (Ma et al., 2015), and the voices were from the (International Dialects of English Archive [IDEA], 2011). In the audio recordings from the International Dialects of English Archive (2011), the speakers all read from one of two standard passages: one about a veterinary nurse and one about rainbows. The faces were organized into pairs of strongly and weakly phenotypical faces with similar age and attractiveness based on the normed ratings from the Chicago Face Database and confirmed by the results from our pilot. We matched the 12 face pairs with the 12 voices based on perceived age to ensure that the pairs would be believable. The face pairs were randomly assigned to be viewed while participants listened to only a predetermined portion (22–40 s) of a strongly or weakly stereotypical voice, such that participants saw six of the face pairs with a strongly stereotypical voice and the remaining six with a weakly stereotypical voice. The order of the voices and the side on which the high phenotypicality face appeared (left or right) were counterbalanced to account for any order effects.

##### Manipulation Checks

To ensure participants were paying attention, we asked them to indicate the race of the faces they viewed from a list of six races/ethnicities: White/Caucasian, Black/African American, Hispanic, Latino/a, or Spanish origin, Native American, Asian American, or Native Hawaiian, Samoan, or other Pacific Islander. Participants could also indicate “Other” and enter their own label. Participants were also asked to select the task they completed from a list of four possible tasks: “Rated the attractiveness of faces,” “Identified a speaker’s face from an audio clip,” “Listened to audio clips and chose what race the speaker is,” or “Wrote about stereotyping I have experienced.”

##### Face Choice

Participants indicated which face was likely the speaker’s using radio buttons under the faces. We recoded these values for analyses (0 = Low phenotypicality, 1 = High phenotypicality).

##### Confidence

Participants indicated their confidence in their choice using a slider bar (0% = No confidence, 100% = Complete confidence).

##### Social Attitude Scales

Participants completed a series of measures designed to assess their beliefs about the criminal justice system, racial bias against Black Americans, their own racial identity, and likelihood of engaging in desirable responding.

###### Pretrial Juror Attitude Questionnaire

The Pretrial Juror Attitude Questionnaire (PJAQ; Lecci and Myers, 2008) is a 29-item scale evaluating pretrial juror attitudes and beliefs about the criminal justice system, with six subscales evaluating conviction proneness, system confidence, cynicism toward the defense, social justice, racial bias, and innate criminality. Given the stereotypes associating Black Americans and criminality, only the racial bias subscale (four items) was included in analyses. Due to experimenter error, each item was measured on a 6-point Likert-type scale (1 = strongly disagree, 6 = strongly agree) rather than the intended 5-point scale. Higher scores denote more bias against minorities related to the criminal justice system. Although the total measure showed good reliability, Cronbach’s α = 0.93, the subscale showed poorer reliability (see Table 3).

**TABLE 3:** Means, standard errors, and reliability for the social attitude scales used in Experiment 2.

Measure	*M*	*SEM*	Cronbach’s α
Pretrial juror attitude questionnaire—racial bias subscale	12.7	0.31	0.513
Symbolic racism scale	17.19	0.40	0.816
Collective self-esteem scale—race specific version	67.31	1.42	0.808
Balanced inventory of desirable responding (short form)			
Self-deceptive enhancement	33.12	0.85	0.820
Impression management	32.70	0.83	0.800

###### Symbolic Racism Scale

The Symbolic Racism Scale (SRS; Henry and Sears, 2002) is an 8-item measure used to assess more symbolic or subtle racism against African Americans. Items are measured on a 4-point scale with the exception of item 3 (“Some say that black leaders have been trying to push too fast. Others feel that they haven’t pushed fast enough. What do you think?”), which is measured on a 3-point scale. Total scores can range from 8 to 31, with higher scores indicating higher bias against African Americans.

*Symbolic Racism Scale Collective Self-Esteem Scale*—*Race Specific Version*. The Collective Self-Esteem Scale—Race Specific Version (CSE-R; Luhtanen and Crocker, 1992) is a 16-item scale assessing a person’s own racial or ethnic identity, with four subscales assessing their racial/ethnic membership, their personal view of their racial/ethnic memberships, how others view their racial/ethnic memberships, and how important group membership is to their identity. Each item is measured on a 7-point scale (1 = strongly disagree, 7 = strongly agree). Total scores on the CSE-R can range from 16 to 112, with higher scores showing more collective self-esteem.

###### Balanced Inventory of Desirable Responding

The Balanced Inventory of Desirable Responding short-form (BIDR-16; Hart et al., 2015) is a 16-item scale that assesses socially desirable responding, with two subscales: self-deceptive enhancement and impression management, which reflect unintentional and intentional socially desirable responding, respectively. Traditionally, each item is measured dichotomously; however, Stöber et al. (2002) recommend using continuous scoring to improve reliability and validity. Thus, we assessed each item using a 7-point scale (1 = not true, 7 = very true). For both subscales, total scores can range from 8 to 56, with higher scores denoting greater self-deceptive enhancement and impression management, respectively.

#### Procedure

After indicating their informed consent, participants filled out demographic information and completed an audio check. Next, participants were told a cover story that the research team was interested in understanding how well people can identify a speaker’s face from an audio recording. Participants were told that they could see either White or Black faces in the study, although only Black faces were used. Participants listened to either a strongly or weakly stereotypical voice before choosing which of a pair of faces taken from the pilot was more likely to be the speaker and indicating their confidence in their decision. Participants made 12 two-alternative forced choice decisions and 12 confidence ratings in total. Finally, all participants answered the manipulation checks and completed the social attitude scales before being debriefed and thanked for their work.

### Results

#### Data Analysis

To explore how perceived speech stereotypicality influences face selections, we first ran a mixed effects logistic regression on participants’ chosen faces (Low or High Phenotypicality). The initial model included voices (Low or High Stereotypicality) as a fixed effect and participants and the individual face pairs entered as random intercepts. We also ran a mixed effects regression on choice confidence with the same fixed and random effects to see if speech stereotypicality had any undue influence on participants’ confidence in their face selections.

We followed up both of these analyses by adding our social attitude scales to control for social attitudes about race as well as desirable responding. The racial bias subscale of the PJAQ and the SRS were included, as well as their interaction terms with voices, to investigate whether racism against minorities related to crime or racism against Black Americans affected which face was selected or moderated the effect of voice stereotypicality on face choices. Work by Stepanova and Strube (2012a) found that implicit bias against Blacks moderated the effect of skin color on ratings of racial typicality, indicating that an individual’s racial bias may affect how individuals are categorized. The CSE-R and two BIDR subscales were added as covariates as previous work has found that higher collective self-esteem can affect how individuals categorize faces (Elliott et al., 2017), and higher socially desirable responding, whether intentional or unintentional, may have influenced participants’ face choices, e.g., in an effort to appear less biased. In all models, the voices variable was sum coded, with Low Stereotypicality serving as the reference group. Each social attitude scale was mean-centered before being added to the models. Significance tests were run by conducting likelihood ratio tests comparing the full model to the model without the predictor of interest for all predictors. Follow-up pairwise comparisons were Bonferroni-adjusted for two tests. The data were imported into R Studio using the haven package (Wickham and Miller, 2019). Data were analyzed using the afex (Singmann et al., 2019), lme4 (Bates et al., 2015), and emmeans (Lenth, 2019) packages.

#### Manipulation Checks

The majority of participants correctly answered our manipulation check questions. For the race of the faces, 88.67% of participants noted the faces were Black/African American. For our question on the task, 85.33% of participants correctly noted they were asked to identify a speaker’s face from an audio recording. Despite 34 participants failing at least one of our manipulation check questions, our pattern of results did not differ when these participants were included in the dataset (see Supplementary Material). Thus, our reported results include the full sample of 150 (*N* = 1,800 choices).

#### Face Selection

**FIGURE 1:**
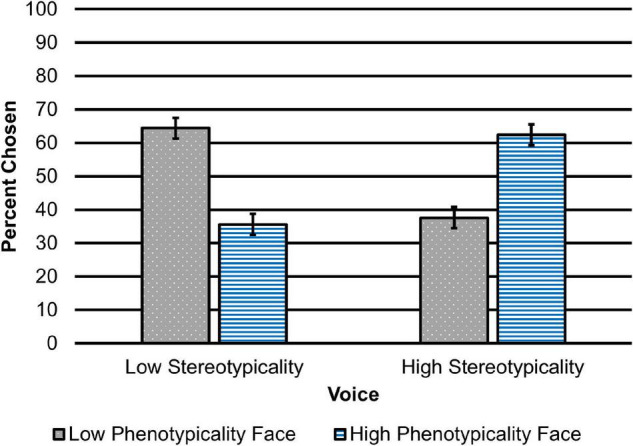
Percent of low and high phenotypicality face selections after listening to low and high stereotypicality speakers. Error bars represent 95% confidence intervals.

We began by examining the results from our model on face choice. As expected, speech stereotypicality influenced participants’ decisions about which face was more likely to belong to the speaker, *B* = −0.60, *SE* = 0.15, *p* < 0.001. Participants who heard the low stereotypicality voice were less likely to choose the high phenotypicality face. After hearing the low stereotypicality voice, participants chose the low phenotypicality face 64.44% of the time and chose the high phenotypicality face 35.56% of the time (Figure 1).

We then looked at the model on choice confidence. Participants’ confidence ratings spanned the range of possible scores (*M* = 66.68, *SEM* = 0.47, Range = 0--100); however, their level of confidence in their decision did not significantly differ by which voice they heard, *B* = --0.47, *SE* = 0.82, *p* = 0.576^2^.

Finally, we looked at the models with the scores from the racial bias subscale from the PJAQ race bias subscale, the SRS, CSE-R, and the two BIDR subscales added (means and SEMs are reported in Table 3). For choice, speech stereotypicality remained a significant predictor, *B* = –0.61, *SE* = 0.16, *p* < 0.001. The interactions between the racial bias subscale and voice and the SRS and voice were also significant, *B* = 0.11, *SE* = 0.02, *p* < 0.001, and *B* = –0.06, *SE* = 0.01, *p* < 0.001, respectively. The slope for racial bias on face choice was greater for low stereotypicality voices than high stereotypicality voices, *z* = 5.71, *p* < 0.001. At 1 SD above the mean on the racial bias subscale, participants were not significantly more likely to choose the high phenotypicality face after hearing the high stereotypicality voice, *z* = 1.22, *p* = 0.442. However, at 1 SD below the mean, participants were over seven times more likely to choose the high phenotypicality face after hearing the high rather than low stereotypicality voice, *z* = 5.85, *p* < 0.001, *OR* = 7.58.

Separately, the slope for racism against Black Americans on face choice was greater for high stereotypicality voices than low stereotypicality voices, *z* = 4.05, *p* < 0.001. At 1 SD above the mean on the SRS, participants were around six times more likely to choose the high phenotypicality face after hearing the high vs. low stereotypicality voice, *z* = 5.19, *p* < 0.001, *OR* = 6.01. At 1 SD below the mean, participants were not significantly more likely to choose the high phenotypicality face after hearing the high stereotypicality voice, *z* = 1.90, *p* = 0.114. No other predictors were significant, *p*s ≥ 0.197.

For confidence, speech stereotypicality remained non-significant, *B* = –0.47, *SE* = 0.81, *p* = 0.575. However, the racial bias subscale of the PJAQ, *B* = 1.87, *SE* = 0.41, *p* < 0.001, and the BIDR self-deceptive enhancement subscale were significant predictors of confidence, *B* = 0.32, *SE* = 0.14, *p* = 0.026. As participants showed greater racial bias and as their own tendency to unintentionally engage in socially desirable responding increased, so too did people’s confidence in their choices. The other predictors were not significant, *p*s ≥ 0.053.

### Discussion

Speech stereotypicality influenced people’s judgments about physical appearance in Experiment 2. Participants were more likely to choose faces that matched the level of stereotypicality (low or high) of the voice they heard. Critically, even when controlling for participants’ social attitudes, the link between speech and face stereotypicality remained. However, participants’ explicit and subtle racism moderated the effect of speech stereotypicality on their face choices. Specifically, as participants expressed more racial bias on the PJAQ, they were less likely to choose the face that matched the level of stereotypicality with the voice they heard. Separately, as participants endorsed more subtle racist beliefs against Black Americans, they were more likely to choose the face that had the same level of stereotypicality as the voice. In other words, participants who endorsed more racist beliefs about minorities and crime appeared less sensitive to the linguistic features when making their face selections, and those who showed more subtle racism against Black Americans appeared more sensitive to those features. The latter finding is consistent with prior work that found those with higher implicit racism against Blacks showed a stronger relationship between skin tone and judgments of race typicality (Stepanova and Strube, 2012a) and suggests that implicit or less direct racial bias influences how individuals categorize others. Why those with more racist beliefs related to crime were less likely rather than more likely to discriminate between the faces remains to be determined, but the low reliability of this scale may have been a factor.

The stereotypicality of the voice did not appear to affect their confidence in their judgments, and this pattern remained when the covariates were added. However, the racial bias subscale of the PJAQ and the self-deceptive enhancement subscale of the BIDR-16 did influence confidence ratings. Those who indicated more racial bias were more confident in their decisions. As the other racism measure was not a significant predictor, it is again unclear what aspect of this racial bias subscale is related to confidence. Those who indicated a greater tendency to engage in unintentional socially desirable responding were also more confident in their decisions, likely reflecting their desire to respond in an overly positive manner (Hart et al., 2015).

From these findings, it appears that how a speaker sounds activates certain expectations about what that speaker should look like, and that these expectations persist regardless of a listeners’ self-reported attitudes about Black Americans, their own racial or ethnic identity, or their propensity to respond in a socially desirable manner.

## General Discussion

Individuals are not evaluated in a vacuum. Rather, people use multiple cues to classify individuals into social categories, which can then activate assumptions about that person’s abilities, traits, and social status. Individuals seen as more typical members of their given social group are more strongly associated with stereotypes about that group (Blair et al., 2002; Walker and Wänke, 2017). Most studies exploring the relationship between how stereotypical an individual is perceived to be and the activation of stereotypes focus on stereotypicality in terms of physical features. We demonstrated that language is another important source of social category information that informs stereotypicality judgments. In two experiments, we investigated whether individuals who sound more stereotypically Black are also more likely to be seen as more stereotypically Black in terms of character traits (Experiment 1) and in terms of their appearance (Experiment 2). Individuals perceived by listeners as sounding more stereotypically Black were associated with more stereotypical traits about Black Americans and with more phenotypically Black faces. Importantly, these findings suggest that speech stereotypicality may operate similarly to phenotypicality, as the linguistic features associated with Black Americans appear to activate stereotypes about members of that group without the listener’s knowledge of the actual race of the speaker.

In Experiment 1, we extended work on perceived stereotypicality and the activation of stereotypical traits from the domain of phenotypicality to speech stereotypicality. Speakers who were rated as sounding more stereotypically Black were assigned a greater proportion of stereotypical traits associated with Black Americans than those who were rated as sounding less stereotypically Black. These results are in line with previous work that greater use of AAVE features, which may be comparable to sounding more stereotypically Black, led to lower ratings of attractiveness and social status (Rodriguez et al., 2004). Our finding also suggests that poorer ratings of speakers who use more AAVE features may be related to a greater activation of stereotypes about Blackness, many of which are negative (e.g., criminal, aggressive, uneducated, lazy).

We also found evidence that this relationship between race, speech stereotypicality, and assigned stereotypic traits only applied to those who were perceived as both Black and North American. This finding, along with the fact that our U.S.-based listeners were less likely to identify UK speakers as Black and did not categorize our Black speakers with 100% accuracy, is consistent with the expectations of the Dialectal-Race Hypothesis; that is, to categorize speakers, listeners rely on their cultural knowledge of how a group of speakers talk (Perrachione et al., 2010). If our listeners were relying only on acoustic features caused by anatomical differences that may exist between groups, we would expect that listeners would have no problems correctly identifying the race of our Black speakers and that perceptions of “sounding stereotypically Black” alone, regardless of country of origin, would be sufficient to activate stereotypes about Black Americans. Instead, our results suggest that listeners’ knowledge of dialects influenced how they attributed traits to stereotypical-sounding Black speakers from different regions.

In Experiment 2, speech stereotypicality activated expectations about phenotypicality, such that strongly stereotypical Black voices were associated with more phenotypically Black faces, and weakly stereotypical Black voices were associated with less phenotypically Black faces. While some physical characteristics do influence how speakers sound, such as height and weight (Krauss et al., 2002) or age-related changes to the structure of the vocal tract (Caruso et al., 1995), many of the associations between voice and appearance are informed by the surrounding cultural context. Given that there is no inherent reason why more stereotypical-sounding Black speakers should also look more stereotypically Black, the results from Experiment 2 suggest that the linguistic cues that listeners have associated with being more stereotypically Black activate expectations about the speaker’s appearance.

Notably, while anti-Black bias can influence face identifications (e.g., when choosing between faces with lighter or darker skin; Alter et al., 2016), the relationship between voice and face stereotypicality persisted regardless of participants’ social attitudes. As is true with physical Afrocentric features, listeners appear unaware of the influence of linguistic features on their judgments. Blair and colleagues (Blair et al., 2002, 2004b) found that individuals relied on Afrocentric features independently from race to assign stereotypic traits, and that although they could suppress the influence from race, they could not suppress the influence of Afrocentric features, even when they were instructed to. Further, their inability to suppress the influence of Afrocentric features was not due to ignorance, as individuals were able to identify Afrocentric features when asked. Linguistic cues associated with “sounding Black” could be another example of feature-based stereotyping. Participants in our studies may have been similarly unaware that they were relying on these cues to assign stereotypic traits or to make face judgments because discrimination against others for their way of speaking is not particularly taboo in American society. It remains to be determined if participants could suppress the influence of linguistic features when made aware of them. More conclusive research on this topic is needed to determine whether linguistic features are used for feature-based stereotyping, particularly to establish whether these cues operate independently from race, as all of the speakers in our study were from the same racial group.

These results have critical implications about how speakers who sound more stereotypically Black may be perceived and treated by others. Previous work has found that those who use AAVE are less likely to have access to housing compared to those using General American English (Purnell et al., 1999; Massey and Lundy, 2001), and those who can be identified as Black from speech earn 12% less than their White counterparts, even when controlling for skill, family background, and schooling (Grogger, 2011). How speech patterns influence such biases, particularly wage discrimination, is unknown, but stereotypicality is likely a contributing factor. Concerningly, given the association between Blackness and criminality, speakers who sound more stereotypically Black may also face similar biases within the legal system. Mock courtroom studies have previously found that in some circumstances defendants who are Black and use a non-standard dialect are judged more harshly than those who use a more standard dialect (Dixon et al., 2002; Cantone et al., 2019; cf. Kurinec and Weaver, 2019). However, whether the perceived stereotypicality of the speaker moderates this effect and whether it influences other judgments, such as those made by law enforcement, judges, and eyewitnesses, remains an area for investigation.

That speech stereotypicality could influence decisions in the legal system despite people’s awareness of anti-Black discrimination in the field is not entirely unfounded, as stereotypical features have previously been shown to affect these types of judgments. More phenotypically Black individuals are more likely to be misidentified by eyewitnesses (Knuycky et al., 2014; Kleider-Offutt et al., 2017), particularly when other Black stereotypes (e.g., drug dealer) are activated (Kleider et al., 2012; Osborne and Davies, 2013). Additionally, regardless of race, those with more Afrocentric features are more likely to receive longer criminal sentences (Blair et al., 2004a). Speech stereotypicality may operate in a similar manner. For instance, individuals who sound more stereotypically Black may be expected to appear more phenotypically Black, biasing eyewitness’s memory for the individual and leading to misidentifications. Separately, those who sound more Black may directly activate negative stereotypes about Black Americans, impacting identifications and legal judgments. Future research is needed to determine how speech stereotypicality may influence eyewitness identifications and other forms of legal decision-making.

Although this work provides initial evidence that speech stereotypicality activates racial stereotypes, several topics warrant further investigation. First, both experiments in this study used predominantly non-Black samples, with the majority of participants identifying as White. While it is possible that using such a sample may have exacerbated out-group biases, strengthening the relationships between speech stereotypicality and stereotypical traits or more phenotypical faces, previous work has found that Black Americans often report similar perceptions of AAVE as White Americans, rating the use of AAVE more negatively than standard American English (Doss and Gross, 1992; Payne et al., 2000; McCluney et al., 2021). Further, previous work looking at the stereotypes associated with light- and dark-skinned Blacks found that both White and Black participants associated more stereotypical Black traits with darker-skinned Blacks (Maddox and Gray, 2002). Given that darker skin is seen as more phenotypically Black, it is likely that a sample of only Black Americans would similarly associate more Black stereotypes—to include stereotypes about appearance—with those perceived as sounding more stereotypically Black. However, it is important to note that there are instances when Black Americans express more favorable opinions of AAVE, such as in less formal community settings or when the individual has greater commitment to their Black identity (White et al., 1998; Koch et al., 2001; Rahman, 2008). Thus, whether these effects would replicate in an all-Black sample may depend on how formal the participants perceive the setting and the strength of their identification as members of the Black community.

Second, the experiments in this study intentionally included multiple speakers using different dialects at differing degrees, both to capture more natural speech utterances and, in Experiment 1, to provide listeners with a range of speakers to evaluate. Had we utilized a handful of speakers switching between a more or less stereotypical-sounding style of speech, we could have risked speakers sounding unauthentic or forced, impacting our ratings (Garrett, 2010; Guy and Cutler, 2011). However, if at all possible, future work should try to find speakers who are sufficiently proficient in shifting their degree of AAVE or similar dialect use. This would allow researchers to implement the matched-guise technique, which utilizes the same speaker for two or more dialects or languages (Lambert, 1967), minimizing the influence of any linguistic variables that are not of interest (e.g., intonation, pace) while maintaining the desired range in dialect strength in an authentic way.

Third, we relied on listeners’ ratings to determine how stereotypical speakers sounded. Listeners have been shown to have similar ideas of the standardness of a given dialect as that of researchers (Schüppert et al., 2015; Nejjari et al., 2019), but it is unclear whether this reliability in ratings extends to perceived stereotypicality. However, what constitutes stereotypically Black speech is likely subjective, depending on an individual listener’s own familiarity with Black speech and their social or cultural knowledge of what does or doesn’t sound stereotypical. It would be beneficial to have a more objective approach to stereotypicality research. For instance, the audio recordings used in stereotypicality research could be analyzed for the linguistic features present to determine whether an increase in the quantity of linguistic features associated with Black Americans leads to an increase in perceived stereotypicality, or if there are specific features that trigger such judgments.

Finally, the speakers in this study varied in the content of their utterances. The speakers in Experiment 1 talked about a variety of topics and spoke in either more casual (e.g., video blog) or more formal (e.g., panel talk) settings. Alternatively, the speakers in Experiment 2 read one of two standard texts. On one hand, the audio used in Experiment 1 likely provided more natural utterances for listeners to evaluate. On the other hand, the audio in Experiment 2 more carefully controlled for the content of the utterances, and previous research suggests that judgments about speakers who sound Black may be moderated by their message content (Johnson and Buttny, 1982). Thus, it would be worthwhile for future research to find a balance between natural sounding utterances and ensuring that the content of said utterances is suitably limited to avoid influencing judgments of stereotypicality.

In sum, this work provides an initial look into the relationship between how stereotypically Black an individual speaker sounds and the activation of stereotypes about character traits and phenotypicality. These findings extend previous work on stereotypicality and suggest that other aspects of an individual beyond physical appearance may serve as cues that inform how stereotypical an individual is believed to be. Since discrimination based on how a person speaks is not explicitly prohibited under U.S. law, understanding how speech stereotypicality contributes to judgments about others is needed to guide the development of regulations and other protections to ensure more equitable treatment for such speakers in housing, employment, and the legal system. Those currently in those fields should be mindful of how speech stereotypicality may be influencing decision-making.

## Data Availability Statement

The datasets presented in this study can be found in online repositories. The names of the repository/repositories and accession number(s) can be found below: https://osf.io/ungrs/.

## Ethics Statement

The studies involving human participants were reviewed and approved by the Baylor University Institutional Review Board. The patients/participants provided their written informed consent to participate in this study.

## Author Contributions

CK and CW contributed to the conception and design of the study. CK oversaw the data collection and analysis and wrote the initial draft of the manuscript. Both authors contributed to the article and approved the submitted version.

## Conflict of Interest

The authors declare that the research was conducted in the absence of any commercial or financial relationships that could be construed as a potential conflict of interest.

## Publisher’s Note

All claims expressed in this article are solely those of the authors and do not necessarily represent those of their affiliated organizations, or those of the publisher, the editors and the reviewers. Any product that may be evaluated in this article, or claim that may be made by its manufacturer, is not guaranteed or endorsed by the publisher.

## Footnotes

1 After collecting data it was noted that in one audio clip, a speaker mentioned eating chicken (Speaker 14), which, given the stereotype about Black Americans and chicken (Demby, 2013), could have potentially primed participants to view this speaker as more Black (see Gaither et al., 2015). However, removing this speaker from analyses did not meaningfully change our pattern of results. As a result, this speaker was left in our final dataset.

2 Adding the counterbalance condition to the base models for choice and confidence did not alter our pattern of results, and counterbalance condition was not a significant predictor in either model, *p*s ≥ 0.235. Therefore, we did not include counterbalance condition in our covariate models.
