# Supplementary material for: “Sounding Black”: Speech Stereotypicality Activates Racial Stereotypes and Expectations About Appearance
Source: Front Psychol. 2021 Dec 24;12:785283. doi: 10.3389/fpsyg.2021.785283 (PMC8740186; doi:10.3389/fpsyg.2021.785283)
Supplement: Supplementary file 1 [file Data_Sheet_1.docx]

Supplementary Material

# Experiment 2 Pilot

## Method

### Participants

Participants (*N* = 82) were recruited from Amazon’s Mechanical Turk (MTurk). Only U.S.-based workers who had completed at least 100 Human Intelligence Tasks (HIT) and who had HIT approval rates of 90% or greater were allowed to participate in this study. Participants who successfully completed the survey were paid $0.50 for approximately 10 minutes of work. Two participants were removed from analyses for failure to follow instructions, leaving a final sample of 80 (*M*_Age_ = 36.63, Range = 20-82). The final sample was approximately equally split between men and women (53.75% men). Although the sample was predominantly White (67.50%), Black (15.00%), Hispanic and Latino/a (5.00%), Asian (8.75%), and Native American (3.75%) identities were also represented. The most frequent level of education completed was a Bachelor’s degree (40.00%), followed by some college (17.50%), a high school degree or equivalent (13.75%), and a Master’s degree (11.25%). Only one participant indicated they had an education level lower than a high school degree.

### Materials

#### **Faces**

Participants in the faces condition were shown 24 of 48 Black male faces taken from the Chicago Face Database (Ma et al., 2015). Using the normed ratings provided in the database, we selected 24 high and 24 low stereotypicality faces from the databased that were relatively matched on age and attractiveness to facilitate ratings for our pilot.

#### Voices

Participants in the voices condition listened to 11 of 22 recordings of Black American male speakers available online from the International Dialects of English Archive (International Dialects of English Archive, 2011). Participants listened to the recordings, which had specific start and end times (ranging from 22-41s, depending on the speakers’ utterances) to avoid overly taxing participants. Previous research has found that listeners are able to correctly classify a speakers’ race after short, 20s clips (Grogger, 2011), and listeners can identify whether a speaker was White or Black after listening to a one-second clip (Walton and Orlikoff, 1994). A research assistant independently rated the voices to ensure there was a range of stereotypicality in the set.

#### Dependent Variables

After viewing a face or listening to a speaker, participants indicated the perceived race of the face or voice from a list of ethnicities before rating the face or voice on its perceived stereotypicality (1 = Not at all stereotypically Black to 7 = Extremely stereotypically Black), attractiveness (1 = Not at all attractive, 7 = Extremely attractive), and age. Finally, participants in the voice category selected whether General American English, African American Vernacular English, or another dialect they entered best described the voice.

### Procedure

At the start of the study, participants were randomly assigned to rate either faces (*N* = 40) or voices (*N* = 40). All participants indicated their consent before beginning any study procedures. After entering in their demographic information, participants in the voice condition completed a manipulation check to ensure their computer audio was functional. Participants then either viewed faces or listened to voices and completed the ratings for the aforementioned dependent variables. The order of the presentation of the faces and voices was randomized to control for any order effects. After completing the study, participants were debriefed and thanked for their time.

## Results

To decide which faces and voices would be used in our experimental materials, we first ranked the stimuli based on their ratings of stereotypicality. Faces with the highest and lowest stereotypicality ratings were matched on age, resulting in 14 pairs of faces. We conducted paired *t*-tests to ensure that these pairs did not differ significantly on age (*p*s ≥ .248) or attractiveness ratings (*p*s ≥ .124) but did differ significantly in stereotypicality ratings (*p*s ≤ .005). Similarly, the voices with the highest and lowest stereotypicality ratings were chosen, resulting in eight high and eight low stereotypicality voices. From these voices, we first selected those that were accurately identified a majority of the time (> 50%) as either General American English or African American Vernacular English. Because laypersons are unlikely to be familiar with dialects, we chose those voices in the high stereotypicality category that were also the most frequently identified as having a Black speaker (> 65%). This left us with 12 total voices (Low stereotypicality = 6, High stereotypicality = 6). Voices and face pairs were matched on age to create the final 12 face pairs and voice combinations.

# Experiment 2 – Excluding Those Who Failed the Manipulation Checks

## Participants

We removed those participants who failed to correctly answer both of our manipulation check questions, leaving us with a sample of 116 (*N* = 1392 choices).

## Results

We first examined whether the voice participants heard influenced the faces they chose. We ran a mixed effects logistic regression on chosen face (Low or High Phenotypicality) with voice (Low or High Stereotypicality) entered as a fixed effect and participants and the individual face pairs entered as random intercepts. Voice was a significant predictor, *B* = -0.73, *SE* = 0.19, *p* < .001. After hearing the low stereotypicality voice, participants were less likely to choose the high stereotypicality face. After hearing the low stereotypicality voice, participants chose the low phenotypicality face 66.8% of the time and the high stereotypicality face only 33.2% of the time. Including counterbalance condition as a covariate did not change the pattern of results. Speech stereotypicality remained a significant predictor, *B* = -0.73, *SE* = 0.19, *p* < .001, but counterbalance was not significant, *B* = 0.10, *SE* = 0.06, *p* = .114.

We then examined whether the voice participants heard influenced their confidence in their choices. We ran a mixed effects regression on choice confidence with the same fixed and random effects as in the previous model. Voice did not influence choice confidence, *B* = -0.56, *SE* = 0.91, *p* = .549. Once again, adding counterbalance condition did not affect the pattern of results, and counterbalance was not a significant predictor, *B* = -0.71, *SE* = 1.55, *p* = .648.

Finally, we added in our social attitude covariates (mean-centered) to each model to determine whether individual beliefs influenced subjects’ choices or confidence. The racial bias subscale of the PJAQ and the SRS were entered, along with their interactions with speech stereotypicality, as were the CSE-R and the two BIDR subscales.

For choice, voice remained a significant predictor, *B* = -0.71, *SE* = 0.20, *p* < .001. Both the interaction between the racial bias subscale and voice and the interaction between the SRS and voice were significant, *B* = 0.10, *SE* = 0.02, *p* = .001, and *B* = -0.07, *SE* = 0.02, *p* < .001, respectively. A test of simple slopes indicated that the slope of racial bias on face choice was significantly different for low and high stereotypicality voices, *z* = 4.51, *p* < .001, as was the slope of racism against Black Americans, *z* = -4.58, *p* < .001. No other covariates were significant, *p*s > .213.

At 1 SD above the mean on the racial bias subscale, participants were not significantly more likely to choose the high phenotypicality face after hearing the high rather than low stereotypicality voice, *z* = 1.87, *p* = .124, but at 1 SD below the mean participants were nearly nine times more likely to choose the high phenotypicality face after hearing the high stereotypicality voice, *z* = 5.11, *p* < .001, *OR* = 8.89. At 1 SD above the mean on the SRS, participants were around nine times more likely to choose the high phenotypicality face after hearing the high versus low stereotypicality voice, *z* = 5.15, *p* < .001, *OR* = 9.10, but at 1 SD below the mean participants were not significantly more likely to choose the high phenotypicality face after hearing the high stereotypicality voice, *z* = 2.14, *p* = .140.

For confidence, voice was still a non-significant predictor, *B* = -0.57, *SE* = 0.91, *p* = .541, but both the racial bias subscale and the self-deceptive enhancement subscale of the BIDR were significant covariates, *B* = 1.79, *SE* = 0.51, *p* < .001, and *B* = 0.34, *SE* = 0.16, *p* = .040. As participants indicated more racial bias and as their tendency to engage in unintentional socially desirable responding increased, so too did their confidence in their decisions. No other predictors were significant, *p*s > .131.

# References

Grogger, J. (2011). Speech patterns and racial wage inequality. Journal of Human Resources 46, 1–25. doi:10.3368/jhr.46.1.1.

International Dialects of English Archive (2011). International Dialects of English Archive. International Dialects of English Archive. Available at: https://www.dialectsarchive.com [Accessed August 13, 2021].

Ma, D. S., Correll, J., and Wittenbrink, B. (2015). The Chicago face database: A free stimulus set of faces and norming data. Behavior Research Methods 47, 1122–1135. doi:10.3758/s13428-014-0532-5.

Walton, J. H., and Orlikoff, R. F. (1994). Speaker race identification from acoustic cues in the vocal signal. Journal of Speech, Language, and Hearing Research 37, 738–745. doi:10.1044/jshr.3704.738.
